# Supplementary material for: Are Δ9-Tetrahydrocannabinol and Its Major Metabolites Substrates or Inhibitors of Placental or Human Hepatic Drug Solute-Carrier Transporters?
Source: Int J Mol Sci. 2024 Nov 9;25(22):12036. doi: 10.3390/ijms252212036 (PMC11594202; doi:10.3390/ijms252212036)
Supplement: Supplementary file 1 [file ijms-25-12036-s001.zip › ijms-3318608-supplementary.pdf]

Supplementary Information

International Journal of Molecular Sciences

Are Δ⁹-tetrahydrocannabinol and its major metabolites substrates or inhibitors of placental or human hepatic drug solute-carrier transporters?

Xin Chen [1], Zsuzsanna Gáborik [2], Qingcheng Mao [1†], and Jashvant D. Unadkat [1*]

[1] Department of Pharmaceutics, School of Pharmacy, University of Washington, Seattle, Washington 98195, USA
[2] SOLVO Biotechnology, Charles River Laboratories Hungary, Irinyi József u. 4-20, 1117 Budapest, Hungary
* Correspondence: jash@uw.edu; Tel.: +1 (206) 685-2869
† Deceased

Supplementary Table S1: Coefficient of variation (CV% of triplicate determinations) of the uptake of the cannabinoids by placental or hepatic transporters in the absence (DMSO) and presence of their respective inhibitor

OATP2B1	CV%	DMSO			10 µM erlotinib		
	THC	2.9	22.2	12.4	2.2	24.2	7.8
	11-OH-THC	30.4	11.5	11.4	10.0	11.3	4.7
	THC-COOH	13.3	9.7	6.5	8.9	8.1	6.3

OCT3	CV%	DMSO			100 µM corticosterone		
	THC	31.7	9.3	12.3	8.1	5.7	13.9
	11-OH-THC	11.8	17.8	28.1	22.9	11.5	9.5
	THC-COOH	13.1	8.6	18.7	5.5	6.1	8.4

OAT4	CV%	DMSO			200 µM bromsulphthalein		
	THC	29.0	15.2	5.6	23.0	2.2	2.3
	11-OH-THC	21.8	30.0	10.8	12.7	18.4	8.3
	THC-COOH	12.3	6.8	3.7	4.9	7.3	4.4

OATP1B1	CV%	DMSO			500 µM rifampin		
	THC	21.9	20.1	6.7	5.1	11.9	14.7
	11-OH-THC	76.9	57.8	57.1	23.3	45.5	100.0
	THC-COOH	7.9	7.3	17.8	4.9	7.6	4.4

OATP1B3	CV%	DMSO			500 µM rifampin		
	THC	7.8	8.5	79.8	4.3	2.5	80.0
	11-OH-THC	23.1	15.8	26.7	23.1	20.0	23.1
	THC-COOH	4.1	8.1	15.1	8.4	24.0	17.6

OCT1	CV%	DMSO			100 µM quinidine		
	THC	19.5	26.1	10.3	50.5	9.4	13.0
	11-OH-THC	20.0	28.6	14.3	18.8	30.8	18.8
	THC-COOH	13.8	10.9	6.6	37.0	14.7	16.9

OAT2	CV%	DMSO			200 µM ketoprofen		
	THC	7.5	17.1	7.6	41.9	29.2	9.7
	11-OH-THC	14.3	15.4	14.8	18.4	21.4	13.0
	THC-COOH	11.6	21.3	6.0	19.0	32.8	3.3

NTCP	CV%	DMSO			1 µM bulevirtide		
	THC	3.3	8.2	10.7	5.3	9.8	4.5
	11-OH-THC	23.5	14.8	33.3	20.6	12.8	21.4
	THC-COOH	5.6	18.0	10.3	5.3	6.2	23.8

Supplementary Table S2: Coefficient of variation (CV% of triplicate determinations) of the uptake of the prototypic substrates of the placental or hepatic transporters in the absence and presence of their respective prototypic inhibitor (see Table 1) or cannabinoids

CV%	DMSO	Prototypic inhibitor	5 µM THC	0.3 µM 11-OH-THC	2.5 µM THC-COOH
OAPTP2B1	42.4	3.8	27.6	8.5	13.4
	2.7	7.1	20.2	6.2	30.6
	37.3	3.4	10.9	21.2	29.5
OCT3	13.4	47.4	61.8	2.1	4.5
	57.1	95.1	4.4	12.2	5.7
	6.0	13.8	12.5	13.5	15.5
OAT4	68.0	6.8	72.6	15.2	114.0
	15.7	12.7	35.5	13.8	11.9
	2.9	18.5	9.1	21.7	10.0
OATP1B1	3.2	90.5	18.2	9.2	11.4
	16.6	69.1	25.2	30.2	15.0
	3.3	15.3	4.7	12.9	9.3
OATP1B3	3.9	26.5	15.9	4.1	4.1
	3.2	33.1	13.2	4.2	23.5
	12.0	12.5	30.5	14.2	15.8
OCT1	7.9	1.2	12.4	1.6	3.8
	10.7	9.8	20.9	4.5	32.6
	8.9	1.1	43.1	43.9	35.7
OAT2	24.1	5.3	37.1	9.0	8.3
	20.2	8.3	6.3	15.4	3.6
	7.8	3.1	8.8	27.2	16.0
NTCP	7.0	17.6	8.1	12.7	14.4
	9.5	11.1	12.9	3.2	4.7
	10.2	4.9	34.1	42.3	7.6

Supplementary Table S3: Mass spectrometer conditions

Compound	Parent/Daughter (m/z)	Cone (V)	Collision (eV)
THC	315.2900/123.0800	30	22
THC-D_3	318.2900/123.3000	31	20
11-OH-THC	331.2872/193.1584	30	26
11-OH-THC-D_3	334.2872/196.2826	30	26
THC-COOH	345.2872/299.2664	30	20
THC-COOH-D_3	348.2872/302.2859	30	20

The mass spectrometer was operated in the positive atmospheric pressure chemical ionization mode.

Supplementary Table S4: LC-MS/MS gradient

Time (min)	Gradient A%
0.0	90
0.5	90
5.0	5
6.0	5
6.1	90
8.0	90

The aqueous (solvent A) and organic (solvent B) phase used were water and acetonitrile containing 0.2% (v/v) acetic acid, respectively. The flow rate was 0.3 mL/min.
